# Technical note on introducing a digital workflow for newborns with craniofacial anomalies based on intraoral scans - part II: 3D printed Tübingen palatal plate prototype for newborns with Robin sequence

**DOI:** 10.1186/s12903-020-01159-7

**Published:** 2020-06-16

**Authors:** A. B. Xepapadeas, C. Weise, K. Frank, S. Spintzyk, C. F. Poets, C. Wiechers, J. Arand, B. Koos

**Affiliations:** 1 Department of Orthodontics, University Hospital Tuebingen, Osianderstr. 2-8, 72076 Tuebingen, Germany; 2 Section “Medical Materials Science & Technology” University Hospital Tuebingen, Osianderstr. 2-8, 72076 Tuebingen, Germany; 3 Department of Neonatology, University Hospital Tuebingen, Calwerstr. 7, 72076 Tuebingen, Germany

**Keywords:** Additive manufacturing (AM), Digital workflow, Vat-polymerization, Cleft lip and palate (CLP), Down’s syndrome (DS), Robin-sequence (RS), Tübingen palatal plate (TPP), Computer-aided design/computer-aided manufacturing (CAD/CAM), Computer-aided impression (CAI), Functional prototyping

## Abstract

**Background:**

Orthodontic treatment of newborns and infants with Robin-Sequence using the Tübingen Palatal Plate (TPP) is a complex procedure that could benefit from simplification through digitalization. The design of the velar extension (spur) and the palatal base determines the success of the treatment. Therefore, a prototype must be produced and inserted under endoscopic supervision in order to determine the appropriate shape, length and position of the spur. This technical note demonstrates a fully digital workflow for the design and manufacturing of a functional TPP prototype, based on an intraoral scan. This prototype can be altered and individualized digitally for each patient. After the shape and position of the spur have been optimized, the prototype is duplicated using a silicone mold. Then the definitive TPP is manufactured and inserted. We aim to present a workflow which facilitates the fitting procedure and does not require a conventional impression or a physical model to create the appliance.

**Methods:**

As described in part I of this series, the intraoral scan is performed using the 3Shape TRIOS3 scanner and its corresponding acquisition software. The virtual model is rendered in the 3Shape ortho appliance designer and the base of the palatal plate is designed in the 3Shape dental designer. The palatal plate and the virtual model are then imported into Autodesk Meshmixer and a standardized spur is positioned and merged with the base. The TPP is exported in Standard Tessellation Language (STL) format and manufactured on a W2P Solflex 170 DLP printer using VOCO VPrint Splint material (MDR Class IIa).

**Results:**

Based on an intraoral scan, the TPP prototype could be successfully manufactured and proved suitable for the patients’ treatment.

**Conclusion:**

The new digital workflow for the design of the TPP can been successfully implemented into daily clinical routine in our facility. Patients could be alleviated from having to undergo conventional impression procedures and fitting of the TPP could be facilitated by producing multiple functional prototypes for endoscopic evaluation. Through rapid prototyping, the expenditure of the fitting process was reduced, which makes the TPP therapy more efficient and accessible to a wider range of clinicians.

## Background

Guided by the advancement of scanning devices, computer-aided design and manufacturing (CAD/CAM) technology, digital manufacturing and treatment planning is becoming increasingly popular in orthodontics. Meanwhile, more and more dental CAD software solutions have found their way into the market, enabling the digital creation of a variety of appliances for patient care. Until now, mostly adult patients have benefitted from this progress, whereas infant care has largely been left untouched by digitalization. Infants undergoing orthodontic treatment often suffer from a variety of craniofacial disorders that sometimes affect the upper airway. Treatment traditionally requires impression procedures using alginate or silicone, which carry a risk of aspiration or ingestion [1]. As intraoral scanning is capable of accurately registering the upper jaw of neonates and infants, it allows clinicians to switch to a fully digital production of appliances [2, 3]. As the scanning procedure is less invasive and can be interrupted at any time, the advantages are obvious. In Part I of this technical note, an easy-to-implement, linear digital workflow for manufacturing simple stimulation plates was presented, where the appliance is created using the Individual Impression Tray module of the Dental Designer (3Shape, Copenhagen, DK) software. Besides stimulation plates, this workflow can also be applied to create palatal plates for patients with cleft lip and palate (CLP) [4]. The next step in the development goes towards more complex appliances, where more than one software solution is required to replace the conventional workflow. This is the case in patients suffering from Robin Sequence (RS), where the Tübingen Palatal Plate (TPP) is used for early treatment as an alternative to surgical methods. RS is characterized by mandibular retrognathia and micrognathia, glossoptosis and upper airway obstruction (UAO); 80–90% of affected patients also have a U-shaped cleft palate. For treatment, a team of neonatologists, orthodontists and maxillo-craniofacial surgeons at our hospital developed the TPP, which consists of a palatal base plate and a velar extension (spur) of individual length that ends just above the epiglottis. Its purpose is to shift the base of the tongue forward, thereby opening the airway in a minimally invasive [5]. It has been shown in numerous studies to improve respiration, oxygen saturation and swallowing function and also aids in early palate closure which has a positive impact on hearing ability of RS patients [6, 7]. In addition to opening the upper airway, TPP therapy induces mandibular growth which might make surgical advancement treatments redundant [8]. After birth, the aim is to start treatment within 48 h. Upon admission, a sleep study (polysomnogram) is performed, to evaluate the severity of obstruction. The results of this study in combination with an intraoral and extraoral orthodontic examination indicates, whether a TPP or a stimulation plate following the Castillo Morales concept will be chosen for treatment. Independently, the intraoral scan (IOS), which is performed upon admission, will be the basis for digitally creating the appliance.

In order to fit the TPP to the patient’s anatomy, a prototype is manufactured after impression taking. The TPP prototype, which contains a standardized spur, is then introduced under endoscopic supervision. If necessary, the spur is adjusted (material is removed or added) until the optimum length, width and angle are found. For the definitive TPP, a threaded wire is incorporated into the spur to increase stability and extraoral retention elements are attached to the base. Finally, the definitive TPP is inserted under endoscopic control to verify that the changes made had the desired effect. In some cases, two or more adjustment cycles have to be performed until the appliance perfectly fits the patient’s anatomy [9].

In part II of this technical note, a complete digital workflow to produce functional TPP prototypes solely based on an intraoral scan is presented. This enables creating two or more prototypes with different spur-configurations at minimum time expenditure, thus speeding up the fitting procedure and reducing the burden on the patient. Despite the evolution of dental CAD software, there is no established digital workflow yet for TPP manufacturing. Switching to a digital design protocol can be seen as the next step in further advancing TPP therapy and making the process more reproducible and available to a wider range of clinicians. Above and beyond any therapeutic long-term benefits, which still have to be quantified, replacing the conventional impression by intraoral scanning is an immediate benefit for the patient. To visualize the digital workflow, we present it here along a patient case.

## Material and methods

After creating a virtual working model and a palatal plate following the workflow presented in Part I, both were saved as an STL file and imported into Meshmixer (Autodesk, San Rafael, CA, USA). The palatal plate was placed onto the model and the spur, obtained from a scanned prototype, was positioned and merged with the palatal plate. The digital TPP-prototype could then be exported and manufactured using additive or subtractive methods. After endoscopically inspecting the fit of the prototype, spur position and length were manually corrected by a dental technician and the definitive TPP was manufactured conventionally by duplicating the modified prototype. Finally, a threaded wire was incorporated into the spur and two extraoral retention elements were added.

### Digitization of the spur

To obtain a digital standard spur, a conventionally manufactured TPP-prototype, which had previously been created in a semi digital workflow on an additive manufactured (AM) model, was digitalized. The model had been printed using the Digital Light Processing (DLP) device D30II (Rapidshape, Heimsheim, Germany) and Freeprint Model Caramel (DETAX, Ettlingen, Germany) as material. After post-processing, a TPP prototype was created with Orthocryl clear (Dentaurum, Ispringen, Germany) using the sprinkling technique. It was then scanned using a standard-scanner (Ortho X, Dentaurum) and scanning software (Ortho XScan, Dentaurum). Due to its reflective surface, scan spray (Helling, Heidgraben, Germany) was necessary to successfully scan the prototype.

**Fig. 1:**
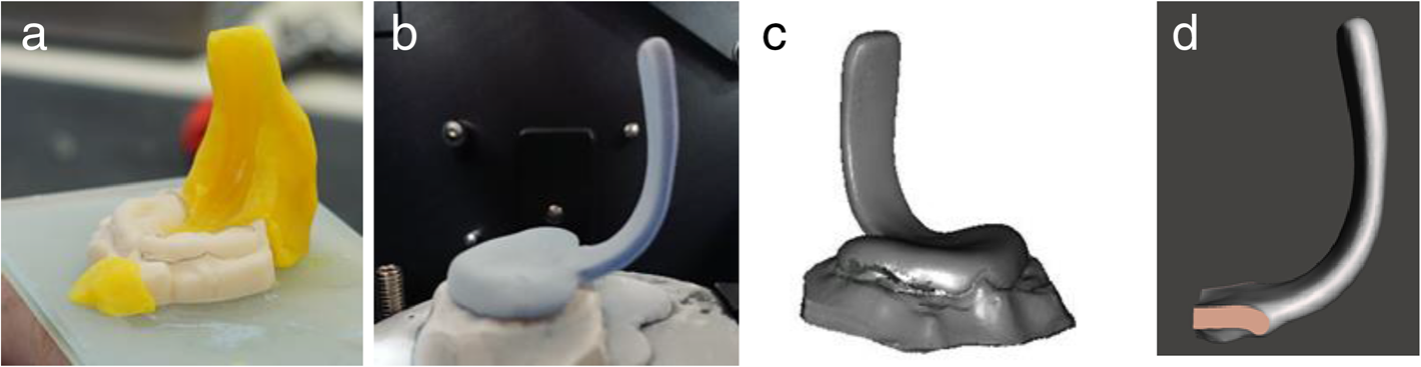
AM Model with Wax mold for the velar extension (**a**), Conventionally manufactured prototype, treated with scanspray, inside the OrthoX-Scanner (**b**), Digitalized TPP (**c**), Isolated Spur-Prototype for digital TPP design (**d**)

The scanned plate was exported as an STL-file and loaded into Meshmixer. Using the plane-cut tool, the scanned spur was separated from the palatal plate by cutting it in three vertical planes. Once digitized, it could be used as a standard spur for prototype production (Fig. 1a-d).

### Intraoral scan

The maxilla was registered using the TRIOS3 intraoral scanner (3Shape, Copenhagen, Denmark) and the TRIOS Scanning Software. Scanning time was 1:16 min where 258 pictures were registered. Before model creation, the raw scan was automatically refined by applying the “postprocessing tool”.

### Design of a Working Model and the palatal plate

**Fig. 2:**
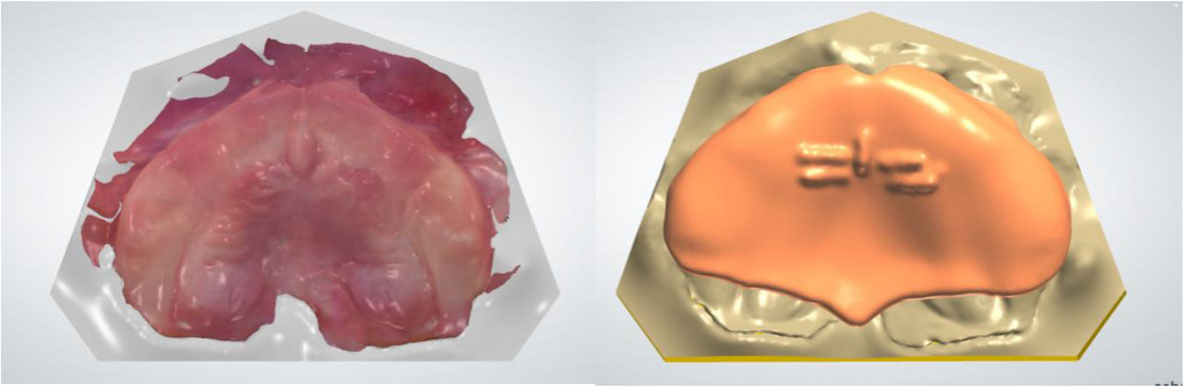
Intraoral Scan on virtual Model (left), palatal plate (right)

Following the procedure described in Part I, first a virtual working model was created using the Model creator module (3Shape), followed by a palatal plate designed using the Individual Impression Tray Module of the Appliance Designer (3Shape) [4]. For the plate design, a thickness of 2 mm was chosen to still allow for grinding out material, in case pressure marks occurred. During model creation, the area of the U-shaped cleft palate could be smoothened and blocked out in freeform to produce a uniform palatal surface and facilitate the design of the palatal plate. In Fig. 2, the working model with the original scan (left) and the palatal plate on the model (right) are displayed. At the posterior end of the plate, a small extension was designed, aligned with the median line for orientation.

### Merging of palatal plate and spur

**Fig. 3:**
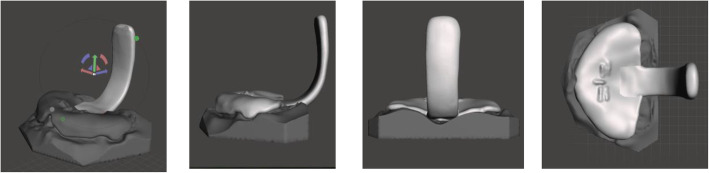
Digital TPP in Autodesk Meshmixer after positioning of the Spur

To assemble the prototype, the virtual model, the designed palatal plate and spur prototype were imported into Meshmixer. After positioning the model with its base onto the horizontal plane, the palatal plate was automatically positioned onto the model by using the align to target function. Now, the digitized spur was positioned onto the posterior end of the palatal plate in relation to the plate, the occlusal and tuber plane of the virtual model as shown in Fig. 3.

**Fig. 4:**
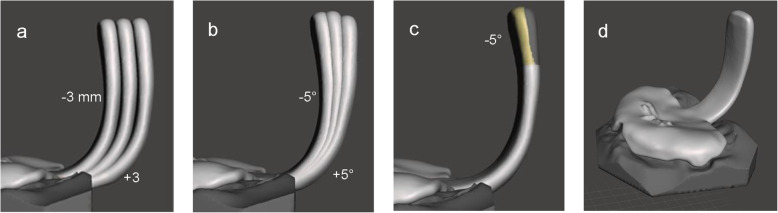
Different spur configurations (**a-c**). TPP after merging plate and spur (**d**)

Before the plate and the spur were unified, the spur could be locked in different positions to create different spur configurations for the initial endoscopy. In Fig. 4, possible configurations of the spur are displayed. The spur was moved from the zero-position 3 mm into an anterior and posterior direction (a), rotated by 5° in anterior and posterior direction (b), and rotated by 5° in anterior direction in the last third of the apical end of the spur (c).

In a final step, plate and spur were merged. First, overlapping areas were removed by manual selection removed. Plate and spur were then selected in the object browser and combined to create one object. The whole surface was again manually selected and the function “Join” was applied to bridge the gap between spur and plate. Finally, the result was smoothened in freeform, mainly at the transition between plate and spur, and an. STL file was created for each of the desired TPP-configurations. In this case, three prototypes were created after Fig. 4a.

### Additive manufacturing

**Fig. 5:**
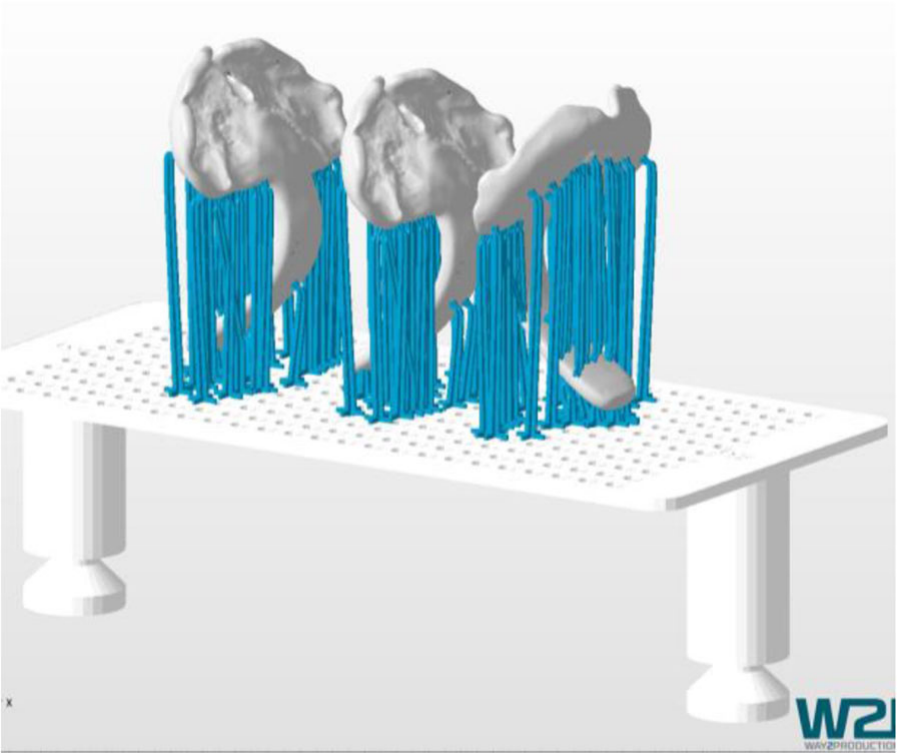
Three TPP prototypes in the manufacturing preparation software. The support structured are depicted in blue color

Manufacturing and postprocessing was performed according to methods published in Part I [4]. TPP-prototypes were printed on the DLP device Solflex 170 (Way2Production, Vienna, Austria) and the material VPrint Splint (VOCO, Cuxhaven, Germany). Layer height was set to 50 μm and the mode “Dental Rapid” was chosen. For printing preparation, Netfabb Premium 2019 (Autodesk, San Rafael, CA, USA) and the support script VOCO Splint 1.0 were used, additional supports were added manually. After manufacturing, the parts underwent postprocessing following manufacturer’s instructions and finished following the procedure described in Part I, which includes the removal of all printing-supports, creating a functional ridge, sanding and polishing the plate. The finished prototypes were then used for the endoscopy. In Fig. 5, the printing-preparation window in Netfabb Premium 2019 and with TPPs on the building platform is displayed. The TPPs were positioned in a way, that no supports had to be placed on the palatal base of the appliance. Supports in this area make post-processing more difficult and could compromise the fit of the appliance onto the palatal area.

### Definitive TPP

**Fig. 6:**
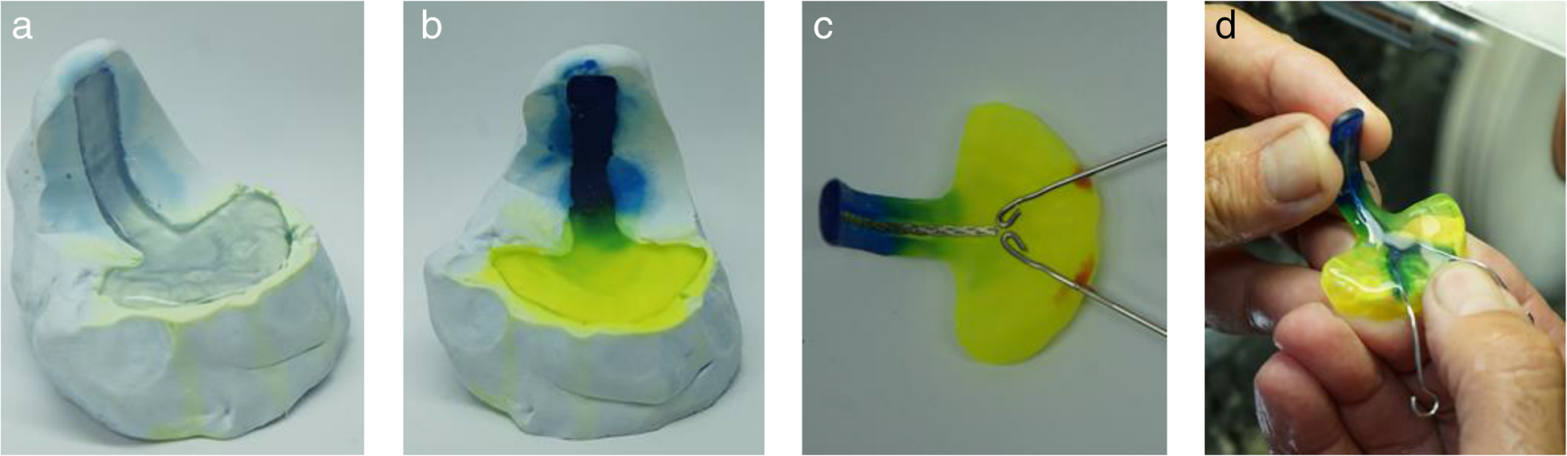
Silicone stencil containing the TPP Prototype (a), mold after filling with Orthocryl (b), polymerized plate with incorporated wires (c), definitive TPP duplicated from the digitally created TPP (d)

Following endoscopy, corrections were made to the AM Prototype and the TPP was duplicated using a silicone stencil and cold polymerizing PMMA (Orthocryl, Dentaurum, Ispringen, D). In this case the spur-configuration with the spur in position 0, showed optimal fit, only the apical end of the spur had to be shorted by 1.5 mm. Theoretically it would be possible to digitally adjust the TPP, but as it is still transferred to a conventional plate, it was felt more efficient to perform adjustments manually at this point. When a satisfying geometry was achieved, wires were polymerized into the duplicated plate and it was polished (Fig. 6).

### Case presentation

A patient with non-syndromic RS is presented with cleft soft palate, snoring, hypoxemia, upper airway obstruction and feeding difficulties. Upon first admission at an age of 2 weeks, a sleep study had been performed in the neonatology department to evaluate the degree of airway obstruction. The examination had resulted in a mixed obstructive apnea index (MOAI) of 7.1/h. The patient was treated with a TPP and tolerated the treatment well. The spur prototype had been manufactured using the above digital workflow and after fitting under endoscopic control. The patient was supplied with a definitive TPP based on the 3D printed TPP. Some adjustment cycles were performed to eliminate pressure marks. The base of the plate was grinded and polished at the corresponding places. Due to the leverage effect of the tongue pressing against the spur, pressure marks can especially occur around the labial frenulum, in the area of the tuber maxillae and the soft palate. To objectify the effect of the TPP, a second sleep study was performed with the definitive TPP, revealing a MOAI of 1.4/h. In addition to TTP therapy, intensive physio- and speech-therapy based on the concept of Castillo Morales was conducted.

## Discussion

Introducing digital workflows into the daily clinical routine of infant care is a gradual process, which started with being able to successfully produce IOS. From here the first stage can be to create conventional appliances on an AM Model, which is another step towards offering a gentler therapeutic concept for patients with orofacial malformations. The workflow presented in Part I of this technical note enabled us to quickly switch from conventionally manufacturing stimulation plates for patients with Down’s syndrome and palatal plates for infants suffering from cleft lip and palate to a fully digital treatment concept extended from scanning to rapid manufacturing (RM) of the appliance.

For patients with RS, the introduction of the TPP concept, as an alternative to more invasive surgical methods, was the first step towards a gentler therapeutic approach and has shown convincing results concerning patient outcome [10]. The digital workflow presented here allowed to improve the fitting procedure and hereby found its way into daily clinical routine where it has proven a feasible enhancement of the conventional procedure. From scan to endoscopy the procedure currently requires about 3 h 30 min. Including manufacturing time.

The reception of the scan by patients and parents was positive and preferred over the conventional procedure. For the clinicians, superiority of scanning is out of question as the procedure can be interrupted at any time. Especially for patients with RS taking a conventional alginate impression can be a life-threatening procedure. Compared to CLP or TS21, scanning is generally more difficult, due to their respiratory problems resulting from their retro-positioned mandible and micrognathia. Intraoral scanning of newborns with craniofacial disorders for appliance design has completely replaced conventional impression taking at our facility. There were no noticeable limitations concerning the acceptance and fit of the digital TPPs compared to conventionally manufactured ones. Rather, the base of the TPP could be manufactured more accurately based on an IOS, resulting in a better fit of the appliance.

After having digitally designed the TPP prototype and receiving an STL (Standard Tessellation Language) dataset of the appliance, there are different means of manufacturing the appliance. In daily clinical routine, additive manufacturing (AM) has great potential for producing a digitally designed TPP, as it is time effective and allows for the exact production of complex geometries. When it comes to choosing a material, it is key to investigate its mechanical properties in comparison with conventional materials to ensure the highest safety standard for the patient. In preparation for switching to a digital TPP, a study on the flexural strength of a Class IIa approved Splint Material (VPrint Splint, VOCO GmbH) was performed. The AM material showed similar flexural strength as conventional cold polymerizing polymer (Orthocryl, Dentaurum) and is therefore suitable for TPP prototype manufacturing [11]. The Material suggested here has been approved to remain in the patient for up to 30 days. As the TPP is removed by the parents daily we are not aware of any biological risks. Despite these encouraging results, a combined approach of digital and manual manufacturing steps was chosen. Therefore, the TPP prototype used for the initial endoscopy is created using the digital workflow and AM. As a wire has to be incorporated for stability, the digital TPP prototype is transferred to the conventional material using duplicating silicone, after correction following endoscopy. Although, the plates occasionally underwent warping when transferring the prototype to the definitive TPP, and therefore did not fit as well onto the palatal area as the printed prototype. This issue could be tackled by manufacturing the TPP in a complete digital workflow involving a definitive TPP created with computer assisted manufacturing. Nonetheless, this does not eliminate the main advantages of the semi-digital approach, i.e. that it allows creation of several prototypes with different spur configurations that can be assessed during endoscopy and are associated with minimal cost. Manufacturing the prototype from clear splint material can make it more difficult to localize the spur during the endoscopy. We are currently reviewing the suitability of colored splint materials for the prototype.

A combination of the 3Shape Dental Designer, 3Shape Appliance Designer and Autodesk Meshmixer proved suitable for creating the TPP. In combination with the virtual model as a reference, it was possible to position the spur in a clinically fitting manner. The base of the TPP fitted well onto the patient’s palate and the spur was visible during endoscopy, which enabled successful fitting of the spur for creating the definitive TPP. However, the design process, as it was performed in this study, requires good knowledge of the CAD-software and can be time consuming depending on the complexity of each case and the quality of the scan. Yet, this is outweighed by the possibility of creating more than one prototype with minimum effort.

## Conclusion

In this technical note, the first complete digital workflow for manufacturing the TPP prototype is presented. After digital design of the TPP, additive manufacturing of the appliance was possible, and the endoscopy could be successfully performed using the digital TPP. The workflow presented here has been successfully applied in > 10 patients, while we have switched to intraoral scanning for all new admissions of infants.

As materials for CAD/CAM technologies in dentistry continuously advance, and a wider spectrum of manufacturing methods becomes available, it should only be a matter of time until the definitive digital TPP can become the new standard of care via rapid manufacturing.

## Data Availability

All data and materials are accessible on a local server of the Department of Orthodontics of the University Hospital Tuebingen, Germany. For further requests please contact the corresponding author.

## Abbreviations

TS21: Trisomy 21/ Down’s Syndrome
CAD/CAM: Computer-Aided Design/Computer-Aided Manufacturing
RS: Robin Sequence
IOS: Intraoral Scanner
UAO: Upper Airway Obstruction
TPP: Tübingen Palatal Plate
AM: Additive Manufacturing
STL: Standard Tessellation Language
RM: Rapid Manufacturing
